# Supplementary material for: Comparative Transcriptomics Analysis for Gene Mining and Identification of a Cinnamyl Alcohol Dehydrogenase Involved in Methyleugenol Biosynthesis from Asarum sieboldii Miq
Source: Molecules. 2018 Dec 3;23(12):3184. doi: 10.3390/molecules23123184 (PMC6321292; doi:10.3390/molecules23123184)
Supplement: Supplementary file 1 [file molecules-23-03184-s001.pdf]

Supplementary Materials for

Comparative Transcriptomics Analysis for Gene Mining and Identification of a Cinnamyl alcohol dehydrogenase Involved in Methyleugenol biosynthesis from *Asarum sieboldii* Miq.

Jinjie Liu [1], Chong Xu [2], Honglei Zhang [3], Fawang Liu[1], Dongming Ma[2,*] and Zhong Liu [1,*]

[1] School of Pharmacy, Shanghai Jiao Tong University, Shanghai 200240 , China; LHJW161903@sjtu.edu.cn (J.L.); fawang90@126.com (F.L.)

[2] Research Center of Chinese Herbal Resource Science and Engineering, Guangzhou University of Chinese Medicine, Guangzhou 510006, China; 15013053420@163.com (C.X.)

[3] Jiusan administration of Heilongjiang farms & land reclamation, Heilongjiang 161441, China; ZHL5555@163.com (H.Z.)

* Correspondence: liuzhong@sjtu.edu.cn (Z.L.); madm@gzucm.edu.cn (D.M.)

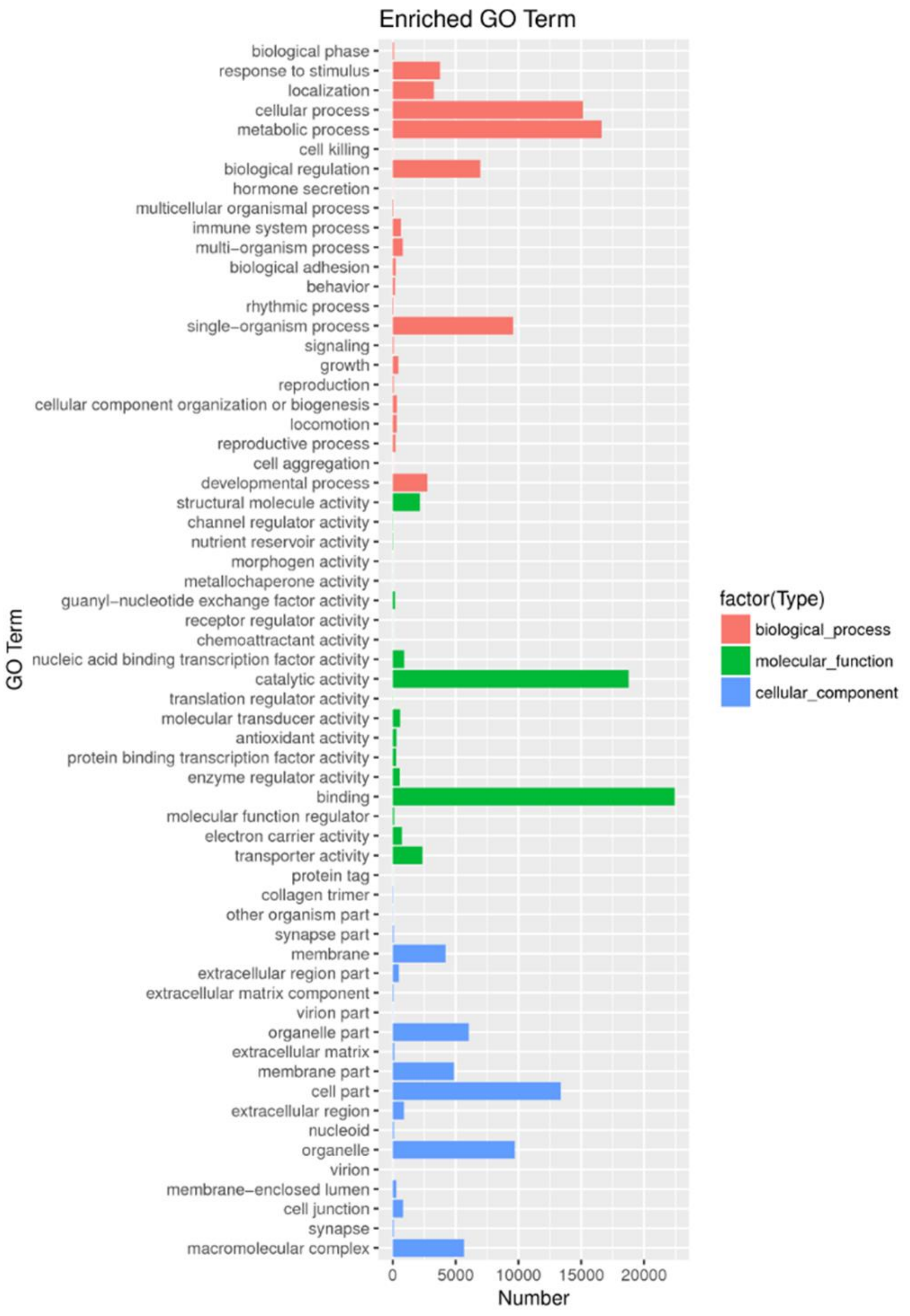

Figure S1. Gene Ontology classification of assembled unigenes.

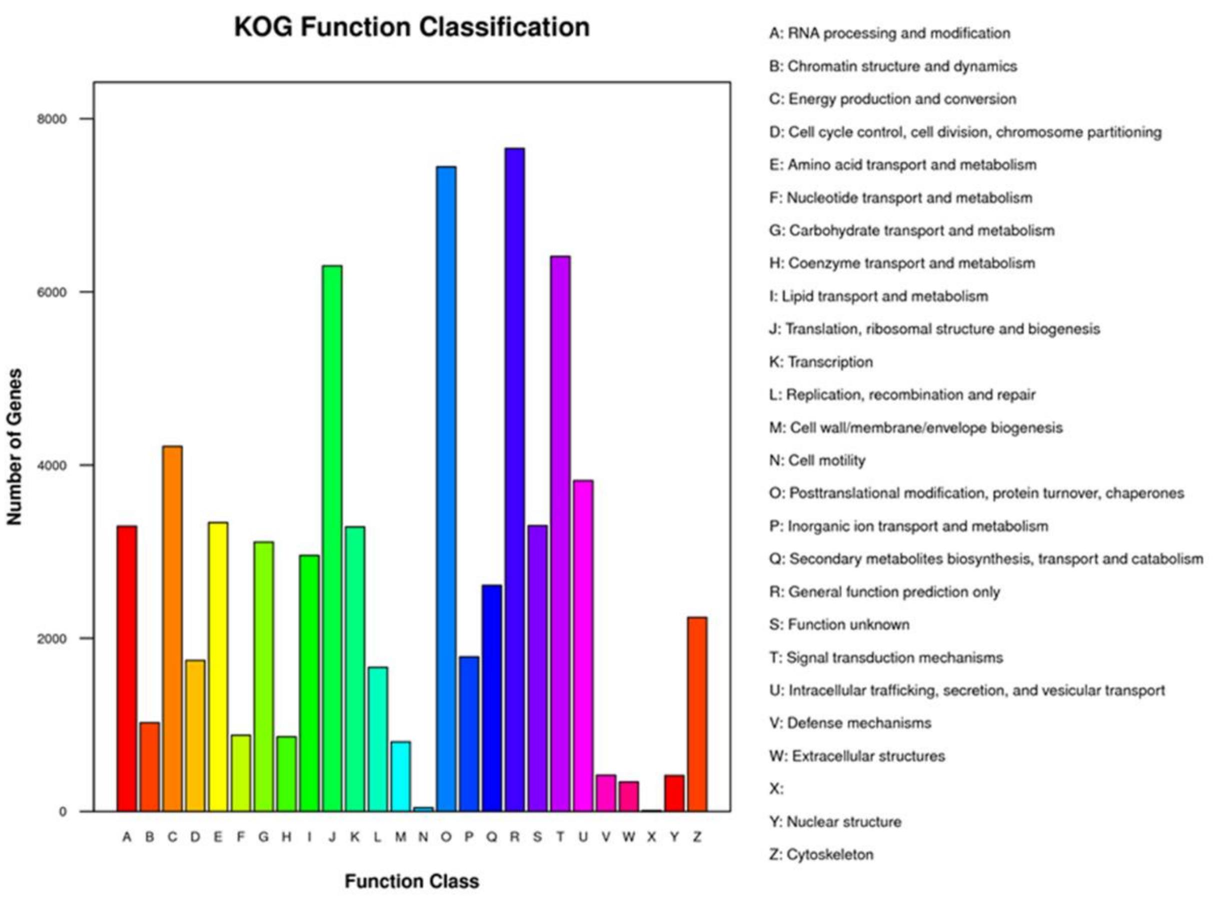

Figure S2. COG function classification of *Asarum sieboldii* Miq.

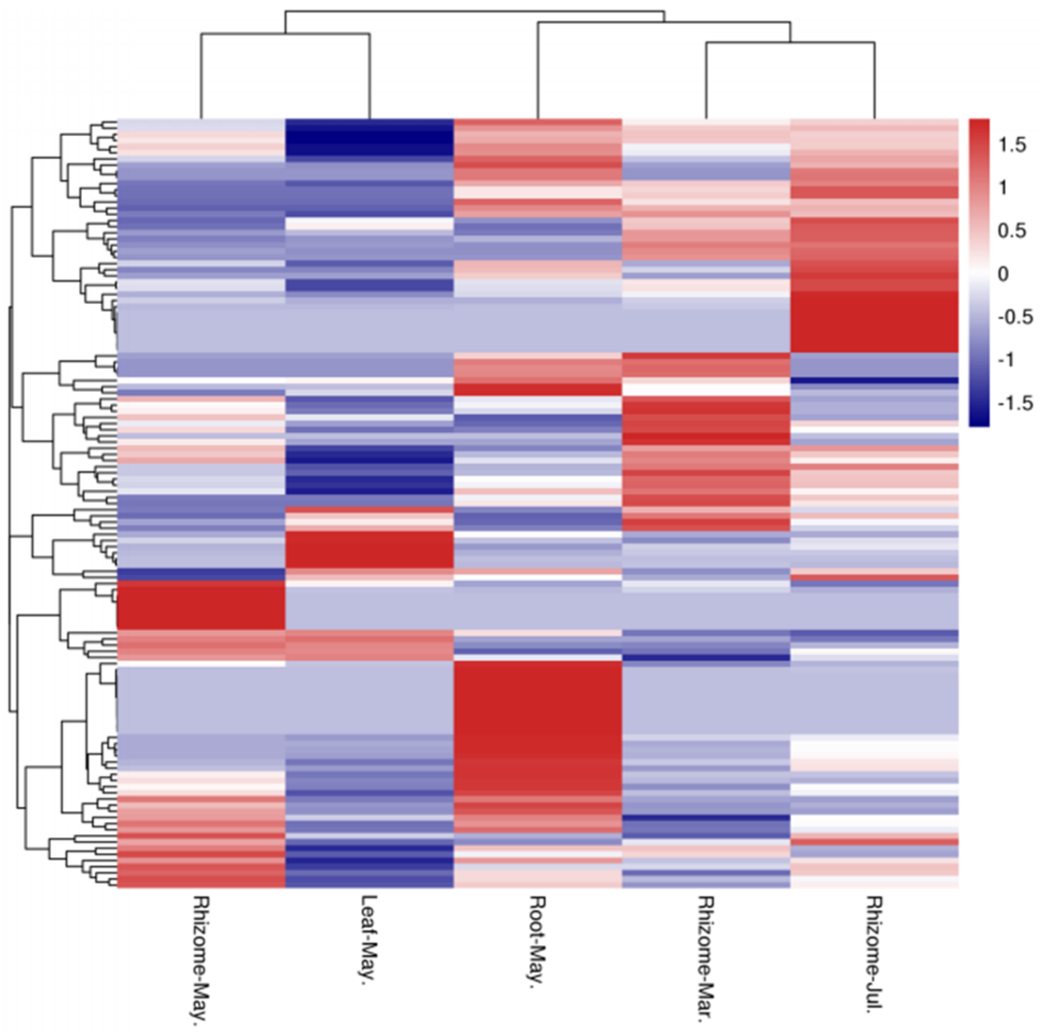

Figure S3. A heatmap of all CYP450s from five samples: root-May, rhizome-May, rhizome-March, rhizome-July and leaf-May.

Table S1. Gene Ontology classification.

Gene Ontology	Class	Total numbers
**Biological process**	metabolic process	16611
	cellular process	15134
	single-organism process	9561
	biological regulation	6958
	response to stimulus	3747
	localization	3266
	developmental process	2736
	multi-organism process	795
	immune system process	637
	growth	435
	cellular component organization or biogenesis	316
	locomotion	311
	biological adhesion	223
	reproductive process	204
	behavior	160
	biological phase	91
	signaling	87
	reproduction	81
	rhythmic process	70
	multicellular organismal process	69
	hormone secretion	17
	cell killing	11
	cell aggregation	4
**Cellular component**	cell part	13355
	organelle	9712
	organelle part	6035
	macromolecular complex	5675
	membrane part	4860
	membrane	4190
	extracellular region	875
	cell junction	810
	extracellular region part	481
	membrane-enclosed lumen	282
	extracellular matrix	110
	nucleoid	89
	synapse	81
	synapse part	80
	extracellular matrix component	71
	collagen trimer	38
	other organism part	22
	virion part	15
	virion	1
**Molecular function**	binding	22439
	catalytic activity	18764
	transporter activity	2365
	structural molecule activity	2166
	nucleic acid binding transcription factor activity	898
	electron carrier activity	723
	molecular transducer activity	584
	enzyme regulator activity	553
	antioxidant activity	291
	protein binding transcription factor	272
	guanyl-nucleotide exchange factor act	162
	molecular function regulator	97
	nutrient reservoir activity	29
	channel regulator activity	25
	translation regulator activity	14
	receptor regulator activity	9
	metallochaperone activity	6
	chemoattractant activity	5
	protein tag	3
	morphogen activity	2

Table S2. Mapping of *Asarum sieboldii* Miq. unique sequences to KEGG biochemical pathways.

KEGG categories represented	No. of uniques
Metabolism	22,194
Amino acid metabolism	2,858
Carbohydrate metabolism	5,085
Nucleotide metabolism	1,246
Lipid metabolism	1,901
Energy metabolism	2,725
Metabolism of cofactors and vitamins	1,018
Metabolism of other amino acids	898
Metabolism of terpenoids and polyketides	414
Glycan biosynthesis and metabolism	652
Biosynthesis of other secondary metabolites	581
Human Diseases	20,725
Infectious diseases: Viral	4,038
Neurodegenerative diseases	3,554
Cancers: Overview	3,499
Infectious diseases: Bacterial	3,416
Genetic Information Processing	11,597
Translation	5,807
Folding, sorting and degradation	3,133
Transcription	1,483
Cellular Processes	7236
Transport and catabolism	2,652
Cell Growth and Death	2,440
Cell Motility	556
Cellular community	1,588
Organismal Systems	13,087
Environmental Information Processing	8,642

Table S3. Primers used in this study.

Name	Primers(5′→3′)	Function
AsCAD-F	ATGAGCAACTCCGATGGTGGA	The clone of AsCAD
AsCAD-R	TCAATGGAGGTTGCTACCT	The clone of AsCAD
AsCAD3-F	ATGACTGAAACGACACCAA	The clone of AsCAD3
AsCAD3-R	CTAGGTCATTGATTGACTG	The clone of AsCAD3
AsCAD5-F	ATGAGTAATCAAAGCGGGAC	The clone of AsCAD5
AsCAD5-R	TCACTTCAAGGAATTCTCA	The clone of AsCAD5
AsCAD7-F	ATGGGTAGCCTTGAGAATGA	The clone of AsCAD7
AsCAD7-R	CTAGATTTTGCTGCGGGCGA	The clone of AsCAD7
pMAL-C5X-AsCAD F	GAAGGATTTCACATATGATGAGCAACTCCGATGGTGGA	The construction of pMAL-C5X-AsCAD
pMAL-C5X-AsCAD R	TACCTGCAGGGAATTCTCAATGGAGGTTGCTACCTGCTACA	The construction of pMAL-C5X-AsCAD
pMAL-C5X-AsCAD3 F	GAAGGATTTCACATATGATGACTGAAACGACACCAA	The construction of pMAL-C5X-AsCAD3
pMAL-C5X-AsCAD3 R	TACCTGCAGGGAATTCCTAGGTCATTGATTGACTG	The construction of pMAL-C5X-AsCAD3
pMAL-C5X-AsCAD5 F	GAAGGATTTCACATATGATGAGTAATCAAAGCGGGACTGATAAT	The construction of pMAL-C5X-AsCAD5
pMAL-C5X-AsCAD5 R	TACCTGCAGGGAATTCTCACTTCAAGGAATTCTCAATATCAATCACGA	The construction of pMAL-C5X-AsCAD5
pMAL-C5X-AsCAD7 F	GAAGGATTTCACATATGATGGGTAGCCTTGAGAATGAGAGAT	The construction of pMAL-C5X-AsCAD7
pMAL-C5X-AsCAD7 R	TACCTGCAGGGAATTCCTAGATTTTGCTGCGGGCGAC	The construction of pMAL-C5X-AsCAD7
qRT-As18S rRNA F	AACGAGACCTCAGCCTGCT	For reference gene As18S rRNA qRT-PCR
qRT-As18S rRNA R	CCCAGAACATCTAAGGGCA	For reference gene As18S rRNA qRT-PCR
qRT-AsCAD F	GCATCATGTGACAGTGATCAG	For AsCAD qRT-PCR
qRT-AsCAD R	CAGTGTCGAGAATGTAGTCG	For AsCAD qRT-PCR
qRT-AsCAD3 F	GCAGCCTCGTGCCTCACCTG	For AsCAD3 qRT-PCR
qRT-AsCAD3 R	GTAGTGGAGCTGCTGCATCC	For AsCAD3 qRT-PCR
qRT-AsCAD5 F	CAGCAGGAGGAGTATTGGA	For AsCAD5 qRT-PCR
qRT-AsCAD5 R	TCAGAGCCAACCTCTTGCAC	For AsCAD5 qRT-PCR
qRT-AsCAD7 F	GGTGTTGCACTGTGGCATCTG	For AsCAD7 qRT-PCR
qRT-AsCAD7 R	CAACTGCCTCGCCTACTCTGA	For AsCAD7 qRT-PCR

Table S4. The accession number(s) of 51 CAD proteins for the phylogeny analysis.

Plant species	Accession number(s)
Arabidopsis thaliana	AtCAD1 - AAP40269
	AtCAD2 - AAP59430
	AtCAD3 - AAP59431
	AtCAD4 - NP_188576
	AtCAD5 - NP_001031788
	AtCAD6 - AAP59428
	AtCAD7 - AAP59432
	AtCAD8 - AAP59433
	AtCAD9 - AAP59429
Aralia cordata	AcCAD1 - D13991
Artemisia annua	AaCAD - ACB54931.1
Asarum sieboldii	AsCAD - AMB21218
	AsCAD3 - ATY69425
	AsCAD5 - ATY69427
	AsCAD7 - ATY69429
Brachypodium distachyon	BdCAD1 - XP_010234665.1
	BdCAD3 - JQ768796.1
	BdCAD5 - JQ768797
	BdCAD6 - XM_003581501.3
Camellia sinensis	CsCAD1 - HQ880207
Cunninghamia lanceolata	ClaCAD1 - JQ904034.1
	ClaCAD2 - JQ904035
Eucalyptus globulus	EgCAD1 - AF038561
Linum usitatissimum	LuCAD1A - DQ487210.1
Lolium perenne	LpCAD1 - AF472591
	LpCAD2 - AF472592
	LpCAD3 - AF010290
Nicotiana tabacum	NtaCAD1 - X62343
	NtaCAD2 - X62344
Oryza sativa	OsCAD1 - AAN09864
	OsCAD2 - ABB04029
	OsCAD7 - CAE05206
	OsCAD9 - AAN05338.1
Picea abies	PaCAD1 - X72675
Pinus radiate	PraCAD - AAC31166
Populus tomentosa	PtoCAD - EU760897.1
	PtoCAD1 - KF145200.1
	PtoCAD8 - KF145195.1
	PtoCAD7 - KJ159966.1
	PtoCAD5 - KF145203.1
	PtoCAD3 - KF145202.1
	PtoCAD2 - KF145201.1
	PtoCAD6 - KF145199.1
	PtoCAD9 - KF145196.1
	PtoSAD - AFZ78656
Populus tremuloides	PtCAD1 - AAF43140
	PtSAD - AAK58693
Sorghum bicolor	SbCAD4 - XP_002462348.1
Triticum aestivum	TaCAD1 - GU563724
Zea mays	ZmCAD1 - AJ005702
	ZmCAD2 - Y13733
